# Bis(3-azoniapentane-1,5-diaminium) cyclo­hexa­phosphate dihydrate: a monoclinic polymorph

**DOI:** 10.1107/S1600536812025172

**Published:** 2012-06-13

**Authors:** Lamia Khedhiri, Samah Akriche, Salem S. Al-Deyab, Mohamed Rzaigui

**Affiliations:** a Laboratoire de Chimie des Matériaux, Faculté des Sciences de Bizerte, 7021 Zarzouna Bizerte, Tunisia; b Petrochemical Research Chair, College of Science, King Saud, University, Riyadh, Saudi Arabia.

## Abstract

In the title hydrated mol­ecular salt, 2C_4_H_16_N_3_^3+^·P_6_O_18_^6−^·2H_2_O, the complete cyclo­hexa­phosphate anion is generated by crystallographic inversion symmetry. The six P atoms of the P_6_O_18_^6−^ anion form a chair conformation and the organic cation has a corrugated linear geometry. In the crystal, the cations and the anions are connected by N—H⋯O hydrogen bonds into slabs propagating in the *ac* plane. The water mol­ecules link the slabs by accepting N—H⋯O links and forming O—H⋯O links. The triclinic polymorph was reported by Gharbi *et al.* [(1995). *J. Solid State Chem.* **114**, 42–51].

## Related literature

For the triclinic polymorph of the title compound, see: Gharbi *et al.* (1995 ▶). For related structures, see: Averbuch-Pouchot & Durif (1991 ▶); Bridi & Jouini (1989 ▶); Kamoun *et al.* (1990 ▶); Khedhiri *et al.* (2007 ▶); Schülke & Kayser (1985 ▶); Khedhiri *et al.* (2003 ▶).
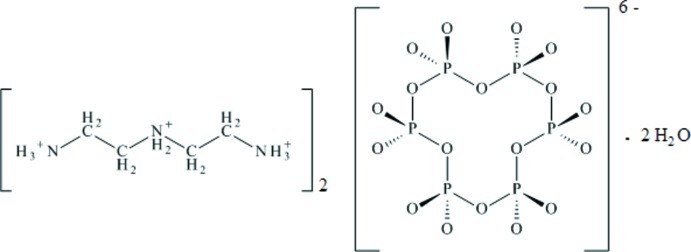


## Experimental

### 

#### Crystal data

2C_4_H_16_N_3_^3+^·P_6_O_18_^6−^·2H_2_O
*M* *_r_* = 722.25
Monoclinic, 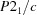

*a* = 10.033 (4) Å
*b* = 16.597 (2) Å
*c* = 8.007 (3) Å
β = 105.07 (2)°
*V* = 1287.6 (7) Å^3^
*Z* = 2
Ag *K*α radiation
λ = 0.56087 Å
μ = 0.27 mm^−1^
*T* = 293 K
0.32 × 0.27 × 0.21 mm

#### Data collection

Enraf–Nonius CAD-4 diffractometer
9091 measured reflections
6303 independent reflections
5211 reflections with *I* > 2σ(*I*)
*R*_int_ = 0.014
2 standard reflections every 120 min intensity decay: 2%

#### Refinement

*R*[*F* ^2^ > 2σ(*F* ^2^)] = 0.034
*wR*(*F* ^2^) = 0.099
*S* = 1.07
6303 reflections
189 parameters
3 restraints
H atoms treated by a mixture of independent and constrained refinement
Δρ_max_ = 0.47 e Å^−3^
Δρ_min_ = −0.97 e Å^−3^

### 

Data collection: *CAD-4 EXPRESS* (Enraf–Nonius, 1994) ▶; cell refinement: *CAD-4 EXPRESS*; data reduction: *XCAD4* (Harms & Wocadlo, 1995 ▶); program(s) used to solve structure: *SHELXS97* (Sheldrick, 2008 ▶); program(s) used to refine structure: *SHELXL97* (Sheldrick, 2008 ▶); molecular graphics: *ORTEP-3* (Farrugia, 1997 ▶) and *DIAMOND* (Brandenburg & Putz, 2005 ▶); software used to prepare material for publication: *WinGX* (Farrugia, 1999 ▶).

## Supplementary Material

Crystal structure: contains datablock(s) I, global. DOI: 10.1107/S1600536812025172/hb6809sup1.cif

Structure factors: contains datablock(s) I. DOI: 10.1107/S1600536812025172/hb6809Isup2.hkl

Additional supplementary materials:  crystallographic information; 3D view; checkCIF report

## Figures and Tables

**Table 1:** Hydrogen-bond geometry (Å, °)

*D*—H⋯*A*	*D*—H	H⋯*A*	*D*⋯*A*	*D*—H⋯*A*
O1*W*—H1*W*1⋯O5	0.84 (1)	2.12 (1)	2.9274 (17)	163 (2)
O1*W*—H2*W*1⋯O2^i^	0.85 (1)	1.85 (1)	2.6938 (16)	175 (2)
N1—H1*B*⋯O1*W* ^ii^	0.89	1.99	2.7817 (15)	147
N1—H1*C*⋯O1	0.89	1.96	2.8054 (14)	159
N1—H1*A*⋯O6^iii^	0.89	1.96	2.8013 (15)	156
N2—H2*A*⋯O8^iii^	0.90	2.10	2.8049 (16)	135
N2—H2*A*⋯O9^ii^	0.90	2.36	3.0891 (15)	138
N2—H2*B*⋯O9	0.90	2.14	2.7973 (16)	130
N2—H2*B*⋯O8^ii^	0.90	2.15	2.8706 (13)	137
N3—H3*B*⋯O5^iv^	0.89	1.99	2.8414 (17)	159
N3—H3*C*⋯O1^v^	0.89	1.93	2.7973 (16)	164
N3—H3*A*⋯O6^vi^	0.89	1.96	2.8008 (16)	157

Symmetry codes: (i) 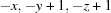; (ii) 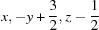; (iii) 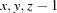; (iv) 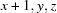; (v) 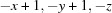; (vi) 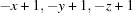.

**Fig. 1.**
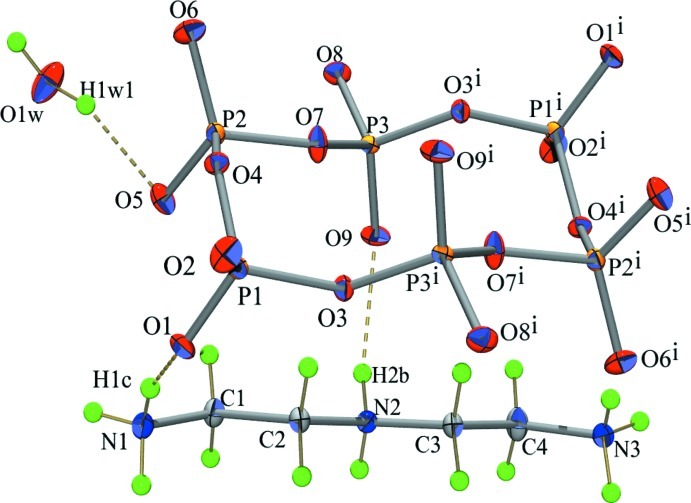
The structure of (I) with displacement ellipsoids are drawn at the 30% probability level. Hydrogen bonds are represented as dashed lines. [Symmetry code: (i) 1 - x, 1 - y, 1 - z.]

**Fig. 2.**
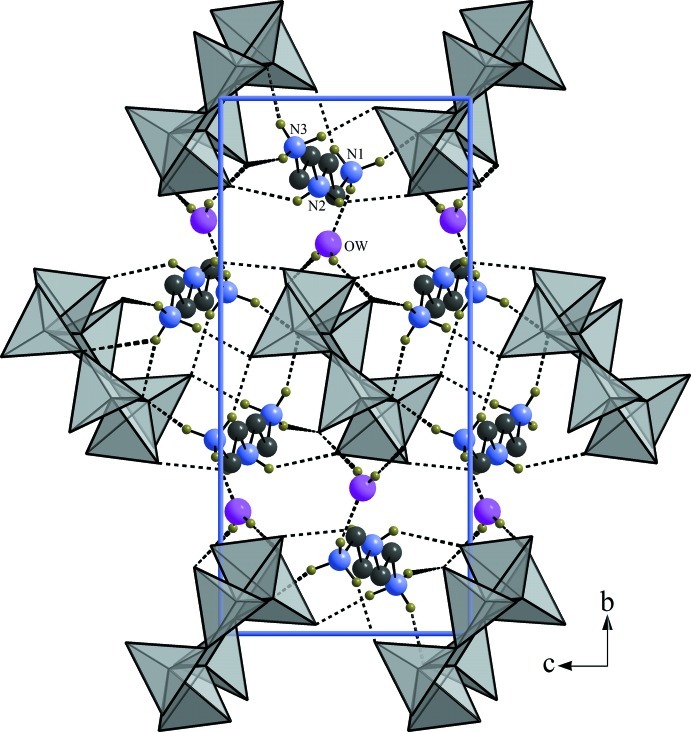
Structure projection of (I) along the a axis. The H-atoms not involved in H-bonding are omitted.

## supplementary crystallographic information

### Comment

The title compound (I), was prepared as part of our ongoing structural studies of inorganic-organic cyclohexaphosphates systems. Its chemical composition includes three entities, P_6_O_18_ ring, water molecules and organic cations [C_4_N_3_H_16_]^3+^ and is polymorphic with a previously described triclinic phase (Gharbi *et al.*, 1995). The geometrical configuration of these entities is depicted in Figure 1, whereas Figure 2 shows the complete atomic arrangement.

The packing of (I) consists of hybrid layers where the organic and inorganic species are alternated. Theses layers, extended perpendicularly to the *b* axis, are also connected between them in the two other directions *via* H-bonds assuring the cohesion of the network. The P_6_O_18_ rings are located around the inversion centers (0, 0, 0) and (1/2, 1/2, 1/2) and are built up by only three independent PO_4_ tetrahedra. The P—P—P angles of 98.85 (1), 109.09 (1) and 138.42 (1)° show that the rings are significantly distorted from the ideal threefold symmetry. It should be noted that these large deviations are commonly observed in cyclohexaphosphates with a ring of low local symmetry (Khedhiri *et al.*, 2003, Khedhiri *et al.*, 2007), as in the title compound. Nevertheless, this distortion is comparatively less important than that observed in Cs_6_P_6_O_18_.6H_2_O, which shows the greatest distortion for the same angles, ranging between 93.2 and 145.5° (Averbuch-Pouchot and Durif, 1991). The great flexibility of the hexamembered P_6_O_18_ rings can probably explain the pronounced distortion observed for the big rings compared with their smaller ring analogues. Examination of the main geometrical features of the three independent PO_4_ tetrahedra (P—O and P–P distances as well as P–O–P or O–P–O angles) shows clearly that, in spite of the P–P–P angles deformation, they are in accordance with values generally observed in condensed phosphate anions.

In the organic entity, N–C and C–C distances and N–C–C and C–N–C angles, spreading within the respective ranges 1.477 (1) - 1.512 (2) Å and 109 (1) -111.5 (9)°, are similar to those observed in others compounds (Kamoun *et al.*, 1990; Bridi *et al.*, 1989). All nitrogen atoms of the used amine are protonated and so it is formulated by [C_4_N_3_H_16_]^3+^. Among the eight hydrogen atoms of this group, only one, H1B, establishes a hydrogen bond with a water molecule, the remaining ones are connected to the external oxygen atoms of three phosphoric rings to form an anionic entity of formula [C_4_N_3_H_16_(P_6_O_18_)_3_]^15-^. The two hydrogen atoms of the water molecule act as a link between successive anions. A three dimensional network of N–H···O and O–H···O hydrogen bonds interconnects the structural arrangement.

This study shows that (I) is a polymorph of the triclinic structure published elsewhere (Gharbi *et al.*, 1995). Both phases have the same chemical formula and some structural analogeous but they present several differences in their crystal data, H-bonding scheme, internal symmetry of the cyclohexaphosphoric anions and particularly the aza-3 pentanediyl-1,5 diaminium which has different conformations where the torsion angles N2–C3–C4–N3, C2–N2–C3–C4, N1–C1–C2–N2 and C3–N2–C2–C1 exhibit the following values -175.34 (9)°, -178.34 (9)°, 171.15 (9)° and 177.79 (9)° in the title compound and 177.67°, 175.32°, 66.45° and -69.92° in the bibliography one.

### Experimental

Single crystals of the title compound were prepared in two steps. In the first one, 50 ml of an aqueous solution of cyclohexaphosphoric acid was prepared by protonation of 4 g of Li_6_P_6_O_18_.6H_2_O, obtained by the Schülke process (Schülke *et al.*, 1985), with an ion-exchange resin (Amberlite IR 120). In the second one, the frech acidic solution (20 ml, 2.6 mmol) was immediately neutralized with a solution of aza-3 pentanediyl-1,5 diamine (2.8 mmol in 10 ml of ehanol) under continuous stirring. Good quality of prismatic-shaped crystals were obtained after a slow evaporation during few days at ambient temperature

### Refinement

All H atoms attached to C and N atoms were fixed geometrically and treated as riding, with C—H = 0.97 Å and N—H = 0.89 Å and with *U*_iso_(H) = 1.2Ueq(C or N).The water H atoms were refined using restraints [O— H = 0.85 (1) A °, H···H = 1.44 (2) A ° and *U*_iso_(H) = 1.5Ueq(O)].

### Crystal data

**Table:**

2C_4_H_16_N_3_^3+^·P_6_O_18_^6^^−^·2H_2_O	*F*(000) = 752
*M**_r_* = 722.25	*D*_x_ = 1.863 Mg m^−^^3^
Monoclinic, *P*2_1_/*c*	Ag *K*α radiation, λ = 0.56087 Å
*a* = 10.033 (4) Å	Cell parameters from 25 reflections
*b* = 16.597 (2) Å	θ = 9–11°
*c* = 8.007 (3) Å	µ = 0.27 mm^−^^1^
β = 105.07 (2)°	*T* = 293 K
*V* = 1287.6 (7) Å^3^	Prism, colorless
*Z* = 2	0.32 × 0.27 × 0.21 mm

### Data collection

**Table:**

Enraf–Nonius CAD-4 diffractometer	*R*_int_ = 0.014
Radiation source: fine-focus sealed tube	θ_max_ = 28.0°, θ_min_ = 2.3°
Graphite monochromator	*h* = −16→3
non–profiled ω scans	*k* = −27→2
9091 measured reflections	*l* = −13→13
6303 independent reflections	2 standard reflections every 120 min
5211 reflections with *I* > 2σ(*I*)	intensity decay: 2%

### Refinement

**Table:**

Refinement on *F*^2^	Primary atom site location: structure-invariant direct methods
Least-squares matrix: full	Secondary atom site location: difference Fourier map
*R*[*F*^2^ > 2σ(*F*^2^)] = 0.034	Hydrogen site location: inferred from neighbouring sites
*wR*(*F*^2^) = 0.099	H atoms treated by a mixture of independent and constrained refinement
*S* = 1.07	*w* = 1/[σ^2^(*F*_o_^2^) + (0.0609*P*)^2^ + 0.1509*P*] where *P* = (*F*_o_^2^ + 2*F*_c_^2^)/3
6303 reflections	(Δ/σ)_max_ = 0.003
189 parameters	Δρ_max_ = 0.47 e Å^−^^3^
3 restraints	Δρ_min_ = −0.97 e Å^−^^3^

### Special details

**Table:**

Geometry. All e.s.d.'s (except the e.s.d. in the dihedral angle between two l.s. planes) are estimated using the full covariance matrix. The cell e.s.d.'s are taken into account individually in the estimation of e.s.d.'s in distances, angles and torsion angles; correlations between e.s.d.'s in cell parameters are only used when they are defined by crystal symmetry. An approximate (isotropic) treatment of cell e.s.d.'s is used for estimating e.s.d.'s involving l.s. planes.
Refinement. Refinement of *F*^2^ against ALL reflections. The weighted *R*-factor *wR* and goodness of fit *S* are based on *F*^2^, conventional *R*-factors *R* are based on *F*, with *F* set to zero for negative *F*^2^. The threshold expression of *F*^2^ > σ(*F*^2^) is used only for calculating *R*-factors(gt) *etc*. and is not relevant to the choice of reflections for refinement. *R*-factors based on *F*^2^ are statistically about twice as large as those based on *F*, and *R*- factors based on ALL data will be even larger.

### Fractional atomic coordinates and isotropic or equivalent isotropic displacement parameters (Å^2^)

**Table:**

	*x*	*y*	*z*	*U*_iso_*/*U*_eq_	
P1	0.20977 (2)	0.430603 (15)	0.26986 (3)	0.01668 (6)	
P2	0.22574 (3)	0.561419 (16)	0.52233 (3)	0.01743 (6)	
P3	0.50730 (2)	0.627794 (15)	0.61516 (3)	0.01533 (5)	
O1	0.14711 (9)	0.48310 (6)	0.11911 (10)	0.02688 (16)	
O2	0.15970 (10)	0.34709 (5)	0.26697 (13)	0.03036 (18)	
O3	0.37237 (8)	0.43405 (4)	0.29127 (10)	0.01950 (13)	
O4	0.19351 (8)	0.47242 (5)	0.44433 (9)	0.01962 (13)	
O5	0.16446 (10)	0.62392 (5)	0.39346 (12)	0.02936 (17)	
O6	0.18261 (11)	0.55876 (6)	0.68613 (12)	0.03256 (19)	
O7	0.38893 (8)	0.56033 (5)	0.56188 (13)	0.02876 (18)	
O8	0.47732 (10)	0.68151 (5)	0.74802 (10)	0.02667 (16)	
O9	0.53563 (11)	0.66399 (6)	0.46030 (11)	0.0340 (2)	
O1W	0.00786 (13)	0.72797 (7)	0.5688 (2)	0.0500 (3)	
H1W1	0.061 (2)	0.6947 (11)	0.540 (3)	0.060*	
H2W1	−0.0488 (19)	0.7066 (13)	0.618 (3)	0.060*	
N1	0.13894 (10)	0.63794 (6)	−0.02369 (13)	0.02486 (17)	
H1A	0.1317	0.6212	−0.1313	0.037*	
H1B	0.0692	0.6709	−0.0228	0.037*	
H1C	0.1363	0.5957	0.0438	0.037*	
N2	0.52234 (9)	0.66493 (5)	0.10700 (11)	0.01944 (14)	
H2A	0.5252	0.6967	0.0169	0.023*	
H2B	0.5298	0.6966	0.2003	0.023*	
N3	0.88883 (10)	0.59139 (7)	0.20294 (12)	0.02576 (18)	
H3A	0.8737	0.5500	0.2663	0.039*	
H3B	0.9692	0.6143	0.2545	0.039*	
H3C	0.8911	0.5739	0.0986	0.039*	
C1	0.27111 (11)	0.68116 (7)	0.04148 (16)	0.02540 (19)	
H1D	0.2726	0.7076	0.1499	0.030*	
H1E	0.2810	0.7220	−0.0411	0.030*	
C2	0.38863 (11)	0.62149 (6)	0.06828 (14)	0.02173 (17)	
H2C	0.3865	0.5857	0.1634	0.026*	
H2D	0.3784	0.5891	−0.0351	0.026*	
C3	0.63924 (10)	0.60786 (6)	0.14047 (14)	0.02126 (17)	
H3D	0.6324	0.5748	0.0387	0.026*	
H3E	0.6344	0.5725	0.2352	0.026*	
C4	0.77604 (12)	0.65144 (8)	0.18517 (19)	0.0315 (2)	
H4A	0.7791	0.6898	0.0948	0.038*	
H4B	0.7874	0.6807	0.2928	0.038*	

### Atomic displacement parameters (Å^2^)

**Table:**

	*U*^11^	*U*^22^	*U*^33^	*U*^12^	*U*^13^	*U*^23^
P1	0.01475 (10)	0.01827 (10)	0.01602 (10)	−0.00204 (7)	0.00221 (8)	−0.00203 (7)
P2	0.01514 (10)	0.01911 (11)	0.01686 (10)	0.00136 (8)	0.00204 (8)	−0.00171 (7)
P3	0.01797 (10)	0.01535 (10)	0.01216 (9)	0.00046 (7)	0.00302 (7)	0.00027 (7)
O1	0.0259 (4)	0.0339 (4)	0.0167 (3)	0.0062 (3)	−0.0019 (3)	0.0011 (3)
O2	0.0304 (4)	0.0226 (4)	0.0406 (5)	−0.0104 (3)	0.0137 (4)	−0.0089 (3)
O3	0.0149 (3)	0.0209 (3)	0.0223 (3)	0.0003 (2)	0.0043 (2)	0.0044 (2)
O4	0.0214 (3)	0.0203 (3)	0.0178 (3)	−0.0036 (2)	0.0063 (2)	−0.0028 (2)
O5	0.0276 (4)	0.0240 (4)	0.0311 (4)	0.0032 (3)	−0.0020 (3)	0.0059 (3)
O6	0.0412 (5)	0.0361 (5)	0.0248 (4)	−0.0009 (4)	0.0165 (4)	−0.0082 (3)
O7	0.0154 (3)	0.0231 (3)	0.0438 (5)	−0.0011 (3)	0.0005 (3)	−0.0097 (3)
O8	0.0383 (4)	0.0210 (3)	0.0211 (3)	0.0043 (3)	0.0083 (3)	−0.0055 (3)
O9	0.0430 (5)	0.0408 (5)	0.0204 (3)	0.0056 (4)	0.0121 (3)	0.0133 (3)
O1W	0.0487 (7)	0.0299 (5)	0.0854 (9)	0.0048 (4)	0.0424 (7)	0.0120 (6)
N1	0.0197 (4)	0.0278 (4)	0.0272 (4)	0.0013 (3)	0.0063 (3)	−0.0007 (3)
N2	0.0197 (3)	0.0188 (3)	0.0203 (3)	0.0004 (3)	0.0059 (3)	0.0006 (3)
N3	0.0182 (4)	0.0354 (5)	0.0225 (4)	−0.0010 (3)	0.0031 (3)	−0.0015 (3)
C1	0.0205 (4)	0.0232 (4)	0.0324 (5)	0.0012 (3)	0.0067 (4)	−0.0026 (4)
C2	0.0188 (4)	0.0215 (4)	0.0241 (4)	0.0003 (3)	0.0042 (3)	0.0004 (3)
C3	0.0183 (4)	0.0207 (4)	0.0242 (4)	0.0011 (3)	0.0044 (3)	0.0010 (3)
C4	0.0209 (4)	0.0270 (5)	0.0436 (7)	−0.0024 (4)	0.0031 (4)	−0.0081 (5)

### Geometric parameters (Å, º)

**Table:**

P1—O2	1.4725 (9)	N2—C3	1.4769 (14)
P1—O1	1.4889 (9)	N2—C2	1.4831 (14)
P1—O3	1.5964 (10)	N2—H2A	0.9000
P1—O4	1.6058 (9)	N2—H2B	0.9000
P2—O5	1.4796 (9)	N3—C4	1.4867 (16)
P2—O6	1.4847 (10)	N3—H3A	0.8900
P2—O7	1.5849 (11)	N3—H3B	0.8900
P2—O4	1.6037 (8)	N3—H3C	0.8900
P3—O9	1.4706 (9)	C1—C2	1.5119 (15)
P3—O8	1.4776 (9)	C1—H1D	0.9700
P3—O7	1.6072 (9)	C1—H1E	0.9700
P3—O3^i^	1.6132 (8)	C2—H2C	0.9700
O3—P3^i^	1.6132 (8)	C2—H2D	0.9700
O1W—H1W1	0.840 (9)	C3—C4	1.5099 (16)
O1W—H2W1	0.849 (9)	C3—H3D	0.9700
N1—C1	1.4783 (15)	C3—H3E	0.9700
N1—H1A	0.8900	C4—H4A	0.9700
N1—H1B	0.8900	C4—H4B	0.9700
N1—H1C	0.8900		
			
O2—P1—O1	117.88 (6)	C2—N2—H2B	109.4
O2—P1—O3	111.74 (5)	H2A—N2—H2B	108.0
O1—P1—O3	105.69 (5)	C4—N3—H3A	109.5
O2—P1—O4	108.01 (5)	C4—N3—H3B	109.5
O1—P1—O4	109.60 (5)	H3A—N3—H3B	109.5
O3—P1—O4	102.90 (4)	C4—N3—H3C	109.5
O5—P2—O6	118.24 (6)	H3A—N3—H3C	109.5
O5—P2—O7	111.58 (6)	H3B—N3—H3C	109.5
O6—P2—O7	110.27 (6)	N1—C1—C2	109.10 (9)
O5—P2—O4	111.65 (5)	N1—C1—H1D	109.9
O6—P2—O4	103.95 (5)	C2—C1—H1D	109.9
O7—P2—O4	99.24 (4)	N1—C1—H1E	109.9
O9—P3—O8	118.73 (6)	C2—C1—H1E	109.9
O9—P3—O7	110.62 (6)	H1D—C1—H1E	108.3
O8—P3—O7	109.68 (6)	N2—C2—C1	109.94 (9)
O9—P3—O3^i^	111.48 (5)	N2—C2—H2C	109.7
O8—P3—O3^i^	108.51 (5)	C1—C2—H2C	109.7
O7—P3—O3^i^	95.29 (5)	N2—C2—H2D	109.7
P1—O3—P3^i^	130.36 (5)	C1—C2—H2D	109.7
P2—O4—P1	132.93 (5)	H2C—C2—H2D	108.2
P2—O7—P3	134.33 (6)	N2—C3—C4	111.46 (9)
H1W1—O1W—H2W1	113.7 (19)	N2—C3—H3D	109.3
C1—N1—H1A	109.5	C4—C3—H3D	109.3
C1—N1—H1B	109.5	N2—C3—H3E	109.3
H1A—N1—H1B	109.5	C4—C3—H3E	109.3
C1—N1—H1C	109.5	H3D—C3—H3E	108.0
H1A—N1—H1C	109.5	N3—C4—C3	108.90 (10)
H1B—N1—H1C	109.5	N3—C4—H4A	109.9
C3—N2—C2	111.02 (9)	C3—C4—H4A	109.9
C3—N2—H2A	109.4	N3—C4—H4B	109.9
C2—N2—H2A	109.4	C3—C4—H4B	109.9
C3—N2—H2B	109.4	H4A—C4—H4B	108.3
			
O2—P1—O3—P3^i^	−28.76 (9)	O6—P2—O7—P3	−82.21 (10)
O1—P1—O3—P3^i^	−158.18 (6)	O4—P2—O7—P3	169.10 (9)
O4—P1—O3—P3^i^	86.88 (7)	O9—P3—O7—P2	−89.73 (10)
O5—P2—O4—P1	50.02 (9)	O8—P3—O7—P2	43.13 (11)
O6—P2—O4—P1	178.57 (7)	O3^i^—P3—O7—P2	154.95 (9)
O7—P2—O4—P1	−67.73 (8)	C3—N2—C2—C1	177.79 (9)
O2—P1—O4—P2	−178.70 (7)	N1—C1—C2—N2	171.15 (9)
O1—P1—O4—P2	−49.08 (8)	C2—N2—C3—C4	−178.34 (9)
O3—P1—O4—P2	63.01 (8)	N2—C3—C4—N3	−175.34 (9)
O5—P2—O7—P3	51.30 (11)		

Symmetry code: (i) −*x*+1, −*y*+1, −*z*+1.

### Hydrogen-bond geometry (Å, º)

**Table:**

*D*—H···*A*	*D*—H	H···*A*	*D*···*A*	*D*—H···*A*
O1*W*—H1*W*1···O5	0.84 (1)	2.12 (1)	2.9274 (17)	163 (2)
O1*W*—H2*W*1···O2^ii^	0.85 (1)	1.85 (1)	2.6938 (16)	175 (2)
N1—H1*B*···O1*W*^iii^	0.89	1.99	2.7817 (15)	147
N1—H1*C*···O1	0.89	1.96	2.8054 (14)	159
N1—H1*A*···O6^iv^	0.89	1.96	2.8013 (15)	156
N2—H2*A*···O8^iv^	0.90	2.10	2.8049 (16)	135
N2—H2*A*···O9^iii^	0.90	2.36	3.0891 (15)	138
N2—H2*B*···O9	0.90	2.14	2.7973 (16)	130
N2—H2*B*···O8^iii^	0.90	2.15	2.8706 (13)	137
N3—H3*B*···O5^v^	0.89	1.99	2.8414 (17)	159
N3—H3*C*···O1^vi^	0.89	1.93	2.7973 (16)	164
N3—H3*A*···O6^i^	0.89	1.96	2.8008 (16)	157

Symmetry codes: (i) −*x*+1, −*y*+1, −*z*+1; (ii) −*x*, −*y*+1, −*z*+1; (iii) *x*, −*y*+3/2, *z*−1/2; (iv) *x*, *y*, *z*−1; (v) *x*+1, *y*, *z*; (vi) −*x*+1, −*y*+1, −*z*.
